# Short-term and long-term outcomes of liver resection for HCC patients with portal vein tumor thrombus

**DOI:** 10.1186/s13578-019-0285-z

**Published:** 2019-03-06

**Authors:** Lei Huo, Wenxin Wei, Zhenlin Yan, Zhengqing Lei, Yanting Xie, Renyan Gong, Shengyu Huang, Ningyang Jia, Yong Xia

**Affiliations:** 1 Department of Hepatic Surgery IV, The Eastern Hepatobiliary Surgery Hospital, Second Military Medical University, 225 Changhai Road, Shanghai, 200433 China; 2 Department of Radiotherapy, The Eastern Hepatobiliary Surgery Hospital, Second Military Medical University, Shanghai, 200438 China; 3 Department of General Surgery, The Zhongda Hospital, Southeast University, Nanjing, 210009 China

**Keywords:** Hepatocellular carcinoma, Portal vein tumor thrombosis, Hepatectomy, Prognosis

## Abstract

**Background:**

Portal vein tumor thrombosis (PVTT) in hepatocellular carcinoma (HCC) is a sign of advanced stage disease, which is associated with poor prognosis. Liver resection (LR) may provide better prognosis in selected patients. In the present study, we aimed to assess information from HCC patients with PVTT who died within 3 months or 2 years after LR in order to identify preoperative factors correlated to short-term or long-term survival, by which inappropriate selection of patients for LR might be avoided in the future.

**Methods:**

A retrospective cohort study consisting of 487 consecutive cases of HCC patients with PVTT was performed from 2008 to 2010 at Eastern Hepatobiliary Surgery Hospital. Medical records, including laboratory values, imaging results and treatment information, were obtained from participants. Study endpoints were survival at 3 months and 2 years post-hepatectomy. Logistic regression analysis was utilized to determine the significant pre-operative factors influencing short-term or long-term survival.

**Results:**

In multivariable analysis, α-fetoprotein, total bilirubin and radiologic ascites were significantly associated with short-term survival, while α-fetoprotein level, clinical significant portal hypertension, extent of PVTT and tumor differentiation were factors significantly associated with long-term survival.

**Conclusions:**

The independent risk factors of poor short-term survival were the liver function-associated, such as factors radiologic ascites and total bilirubin, while tumor differentiation indicating the tumor biology was associated with longer-term survival. In addition, α-fetoprotein was a risk factor associated with both short-term and longer-term survivals.

**Electronic supplementary material:**

The online version of this article (10.1186/s13578-019-0285-z) contains supplementary material, which is available to authorized users.

## Background

Previous study has shown that patients with hepatocellular carcinoma (HCC) and portal vein tumor thrombus (PVTT) have an extremely poor prognosis [1]. A huge proportion of HCC patients (12.5–39.7%) exhibit gross PVTT at the time of diagnosis, while such occurrence of PVTT becomes 44% at the time of death [2]. In addition, the median survival is 2.7–4.0 months when tumor remains untreated [3].

The optimal treatment for HCC patients with PVTT remains controversial. As a standard therapy, sorafenib has been widely used for advanced HCC with PVTT or metastasis. In Asian countries, the median survival is 6.5 months when patients are administered with sorafenib [4]. Many clinical guidelines have shown that hepatectomy is a safe and effective treatment for HCC patients with PVTT if the patients are carefully selected [5–7]. Surgical treatment may offer a chance of cure, and post-operative 5-year overall survival (OS) has been reported to range from 10 to 59% [8–10]. A median survival ranging from 8 to 22 months has been reported for HCC patients with PVTT after surgical treatment [11, 12].

Recently, more aggressive therapeutic schemes, such as operation, interventional radiotherapy, molecularly targeted therapy and other multidisciplinary treatments, have been developed, leading to improved life quality and a prolonged survival time in some patients. As a result of recent advances in surgical techniques and peri-operative management, liver resection (LR) has become a reasonably safe treatment option with an acceptable mortality and morbidity rate [8, 9]. Several tertiary centers have proposed aggressive surgical resection for HCC with vascular invasion [13].

Now, hepatic surgeons may perform palliative surgery in order to improve quality of life, by which the patients can be reasonably to survive for at least 3 months. When patients have a life expectancy < 3 months, such population is generally unsuitable for surgery because the risks and drawbacks, including pre- and post-operative complications, management of post-operative pain, and time required for recovery, usually offset the benefits of intervention, and patients are subsequently referred for systemic therapy or palliative medical care.

In order to recognize which patient will not benefit from surgery, it would be valuable to identify pre-operative factors associated with exceedingly short survival.

In the present study, we aimed to compare clinical presentation and outcomes in patients with HBV-associated HCC, stratified by the presence or absence of cirrhosis and symptoms at time of diagnosis. Patients who died within 3 months of surgery were compared with those long-term survivors (> 2 years). If the patients died within 3 months of surgery, they were inappropriate for surgery. Therefore, we attempted to identify pre-operative factors, which might indicate that anticipated survival does not justify the risk of surgery.

## Results

### Baseline clinicopathological characteristics

**Fig. 1:**
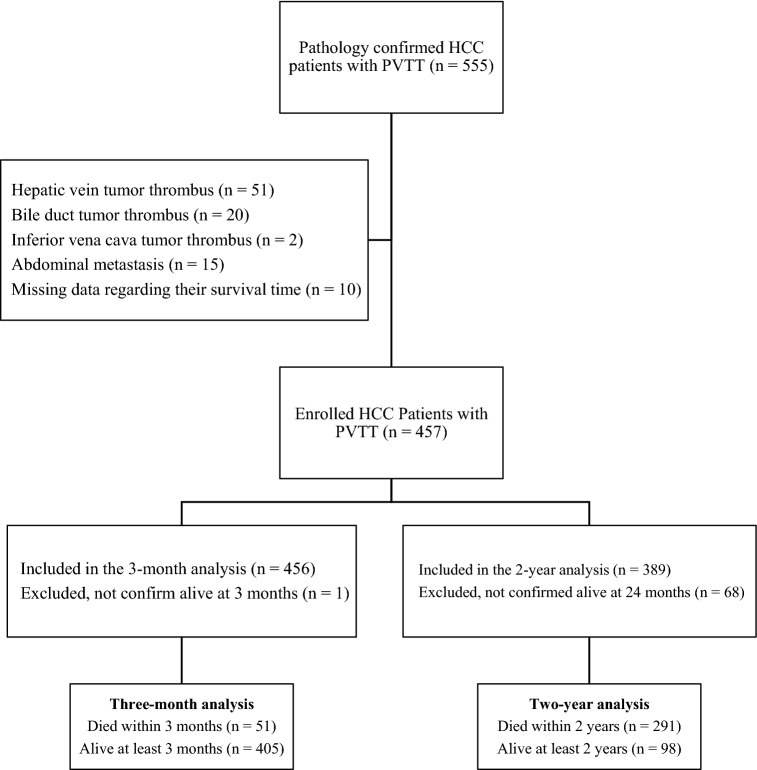
Flow diagram

A total of 457 patients were included in the database. The cohort used in the analysis was under ongoing follow-up. Of the 457 patients, 456 patients were included in the 3-month survival analysis, and 389 patients were included in the 2-year survival analysis (Fig. 1). Univariable and multivariable regression analyses were exclusively conducted on these two smaller groups. The average age of the patients at time of surgery was 47.9 ± 10.3 years, and males accounted for 89.1% of the study population. Table 1 lists detailed baseline characteristics of all patients.

**Table 1:** Baseline characteristics for all patients

Variable	Overall (N = 457)	Alive (n = 139)	Died (n = 318)	*P*
Age, years	47.9 ± 10.3	47.6 ± 9.38	48.1 ± 10.7	0.612
Sex				0.378
Female	50 (10.9%)	12 (8.6%)	38 (11.9%)	
Male	407 (89.1%)	127 (91.4%)	280 (88.1%)	
Viral marker				
HBsAg positive	420 (92.7%)	131 (94.2%)	289 (92.0%)	0.524
Anti-HCV positive	3 (0.7%)	1 (0.7%)	2 (0.6%)	1.000
AFP, μg/L	1210 (1.2–777,600)	1067 (1.2–37,073)	1210 (1.8–777,600)	0.034
AFP, Log 10 μg/L	3.08 (0.08–5.89)	3.03 (0.08–4.57)	3.08 (0.26–5.89)	0.034
TBIL, mg/dL	0.81 (0.26–7.54)	0.77 (0.26–1.87)	0.83 (0.30–7.54)	0.023
ALB, g/L	41.0 (17.2–67.2)	41.3 (17.2–67.2)	40.8 (17.8–65.3)	0.169
PT, s	12.2 (9.6–18.0)	12.2 (10.2–15.2)	12.3 (9.6–18.0)	0.411
ALT, U/L	42.6 (8.7–187.8)	42.1 (11.2–187.8)	42.7 (8.7–169.9)	0.965
PLT, 10^9^/L	156 (25.0–629)	156 (25.0–369)	156 (25.0–629)	0.957
WBC, 10^9^/L	5.51 (1.44–19.4)	5.40 (1.63–11.8)	5.54 (1.44–19.4)	0.187
Hemoglobin, g/L	141 (75.0–195.0)	142 (104.0–174.0)	141 (75.0–195.0)	0.988
Child–Pugh class				0.288
A	448 (98.0%)	138 (99.3%)	310 (97.5%)	
B	9 (2.0%)	1 (0.7%)	8 (2.5%)	
Radiologic ascites				0.019
No	422 (92.3%)	135 (97.1%)	287 (90.3%)	
Mild	35 (7.66%)	4 (2.88%)	31 (9.75%)	
Gastroesophageal varices				1.000
No	382 (83.6%)	116 (83.5%)	266 (83.6%)	
Yes	75 (16.4%)	23 (16.5%)	52 (16.4%)	
Radiologic spleen length, cm	11.4 (6.30–20.2)	11.0 (6.30–17.5)	11.6 (6.30–20.2)	0.060
CSPH				0.237
No	233 (51.0%)	79 (56.8%)	154 (48.4%)	
Mild	183 (40.0%)	48 (34.5%)	135 (42.5%)	
Severe	41 (9.0%)	12 (8.6%)	29 (9.1%)	
Type of hepatectomy				0.177
En bloc resection	289 (63.2%)	81 (58.3%)	208 (65.4%)	
Non-en bloc resection	168 (36.8%)	58 (41.7%)	110 (34.6%)	
Operating time, h	2.0 (0.45–20.8)	2.0 (0.45–20.8)	2.0 (0.45–20.8)	0.175
Hilar clamping time, min	20.0 (0–83.0)	21.0 (0–48.0)	20.0 (0–83.0)	0.248
Intraoperative blood loss, mL	400 (0–45,000)	400 (10–3200)	400 (0–45,000)	0.181
Transfusion				0.229
No	326 (71.3%)	105 (75.5%)	221 (69.5%)	
Yes	131 (28.7%)	34 (24.5%)	97 (30.5%)	
Surgical margins				0.115
R0	363 (79.4%)	115 (82.7%)	248 (78.0%)	
R1	16 (3.5%)	7 (5.1%)	9 (2.8%)	
Unconfirmed	78 (17.1%)	17 (12.2%)	61 (19.2%)	
Extent of PVTT				0.523
Left branch	102 (22.3%)	27 (19.4%)	75 (23.6%)	
Right branch	256 (56.0%)	83 (59.7%)	173 (54.4%)	
Main trunck/contralateral branch	99 (21.7%)	29 (20.9%)	70 (22.0%)	
Tumor number				0.283
Solitary	413 (90.4%)	122 (87.8%)	291 (91.5%)	
Multiple	44 (9.6%)	17 (12.2%)	27 (8.49%)	
Tumor diameter, cm	8.3 (0.4–24.0)	7.5 (0.6–22.0)	9.0 (0.4–24.0)	0.004
Cirrhosis				0.295
No	131 (28.7%)	45 (32.4%)	86 (27.0%)	
Yes	326 (71.3%)	94 (67.6%)	232 (73.0%)	
Tumor differentiation				0.647
II	24 (5.3%)	9 (6.5%)	15 (4.7%)	
III	425 (93.0%)	127 (91.4%)	298 (93.7%)	
IV	8 (1.7%)	3 (2.1%)	5 (1.6%)	

*HBsAg* hepatitis B surface antigen, *anti-HCV* hepatitis C virus antibody, *AFP* α-fetoprotein, *TBIL* total bilirubin, *ALB* albumin, *PT* pro-thrombin time, *ALT* alanine aminotransferase, *PLT* platelet count, *WBC* white blood cell, *CSPH* clinical significant portal hypertension, *PVTT* portal vein tumor thrombus

### Patients who survived < 3 months

Descriptive analysis showed that patients who died within 3 months were significantly associated with a larger diameter, higher bilirubin and higher AFP level. Table 2 displays detailed descriptive analysis of the two groups of patients at 3 months post-hepatectomy.

**Table 2:** Descriptive analysis of survivors versus non-survivors at 3 months

Variable	Alive (n = 405)	Died (n = 51)	*P*
Age, years	47.8 ± 10.2	48.9 ± 10.6	0.453
Sex			1.000
Female	44 (10.9%)	6 (11.8%)	
Male	361 (89.1%)	45 (88.2%)	
Viral marker			
HBsAg positive	370 (92.0%)	49 (98.0%)	0.156
Anti-HCV positive	3 (0.7%)	0 (0.0%)	1.000
AFP, μg/L	1210 (1.2–276,354)	1210 (2.9–777,600)	0.003
AFP, Log 10 μg/L	3.08 (0.08–5.44)	3.08 (0.46–5.89)	0.003
TBIL, mg/dL	0.79 (0.26–7.54)	0.94 (0.34–7.54)	< 0.001
ALB, g/L	40.9 (17.2–67.2)	41.6 (32.4–65.3)	0.799
PT, s	12.2 (9.6–18.0)	12.4 (10.7–18.0)	0.121
ALT, U/L	43.3 (8.7–187.8)	41.3 (9.3–162.1)	0.853
PLT, 10^9^/L	157 (25.0–629)	139 (63.0–358)	0.452
WBC, 10^9^/L	5.51 (1.44–19.4)	5.41 (2.29–10.6)	0.964
Hemoglobin, g/L	141 (94.0–195.0)	146 (75.0–172.0)	0.085
Child–Pugh class			0.266
A	398 (98.3%)	49 (96.1%)	
B	7 (1.7%)	2 (3.9%)	
Radiologic ascites			0.003
No	380 (93.8%)	41 (80.4%)	
Mild	25 (6.2%)	10 (19.6%)	
Gastroesophageal varices			0.397
No	341 (84.2%)	40 (78.4%)	
Yes	64 (15.8%)	11 (21.6%)	
Radiologic spleen length, cm	11.3 (6.3–20.2)	12.0 (8.0–18.9)	0.029
CSPH			0.194
No	212 (52.3%)	20 (39.2%)	
Mild	157 (38.8%)	26 (51.0%)	
Severe	36 (8.9%)	5 (9.8%)	
Type of hepatectomy			0.327
Enbloc	253 (62.5%)	36 (70.6%)	
Non-enbloc	152 (37.5%)	15 (29.4%)	
Operating time, h	2.0 (0.45–20.8)	2.0 (0.45–5.7)	0.862
Hilar clamping time, min	20.0 (0–83.0)	20.0 (0–51.0)	0.474
Intraoperative blood loss, mL	400 (0–45,000)	400 (0–8000)	0.598
Transfusion			0.544
No	291 (71.9%)	34 (66.7%)	
Yes	114 (28.1%)	17 (33.3%)	
Surgical margins			1.000
R0	321 (79.3%)	42 (82.3%)	
R1	15 (3.7%)	1 (2.0%)	
Unconfirmed	69 (17.0%)	8 (15.7%)	
Extent of PVTT			0.281
Left branch	95 (23.5%)	7 (13.7%)	
Right branch	224 (55.3%)	31 (60.8%)	
Main trunck/contralateral branch	86 (21.2%)	13 (25.5%)	
Tumor number			1.000
Solitary	366 (90.4%)	46 (90.2%)	
Multiple	39 (9.6%)	5 (9.8%)	
Tumor diameter, cm	8.0 (0.6–22.0)	10.0 (0.4–24.0)	0.023
Cirrhosis			0.173
No	121 (29.9%)	10 (19.6%)	
Yes	284 (70.1%)	41 (80.4%)	
Tumor differentiation			0.612
II	23 (5.7%)	1 (2.0%)	
III	375 (92.6%)	49 (96.0%)	
IV	7 (1.7%)	1 (2.0%)	

*HBsAg* hepatitis B surface antigen, *anti-HCV* hepatitis C virus antibody, *AFP* α-fetoprotein, *TBIL* total bilirubin, *ALB* albumin, *PT* pro-thrombin time, *ALT* alanine aminotransferase, *PLT* platelet count, *WBC* white blood cell, *CSPH* clinical significant portal hypertension, *PVTT* portal vein tumor thrombus

Patients who had a follow-up visit at, at least, within 3 months or were discharged at this point (n = 405) were compared with those who died within 3 months (n = 51), and results showed the factors associated with death within 3 months in univariable analysis (Additional file 1: Table S1). In multivariable analysis, AFP, per Log 10 μg/L increase (odds ratio [OR], 1.60; 95% CI 1.15 to 2.25; P = 0.010), TBIL, per mg/dL increase (OR, 1.94; 95% CI 1.21 to 3.12; P = 0.026) and positive radiologic ascites (OR, 3.72; 95% CI 1.59 to 8.15; P = 0.009) were the significant factors associated with death within 3 months (Table 3). A total of 51 patients died within 3 months because of systemic progression of cancer (n = 146; 84.4%), complications of spine surgery within 30 days of surgery (n = 7; 4.0%), and unknown or unspecified reasons (n = 20; 11.6%).

**Table 3:** Multivariable logistic regression analysis exploring factors associated with death within 3 or 24 months after hepatectomy

Variable	Three month analysis		Two year analysis	
	Odds ratio (95% CI)	*P*	Odds ratio (95% CI)	*P*
AFP, Log 10 μg/L	1.60 (1.15 to 2.25)	0.010	1.34 (1.06 to 1.69)	0.034
TBIL, mg/dL	1.94 (1.21 to 3.12)	0.026		
Radiologic ascites, positive vs. negative	3.72 (1.59 to 8.15)	0.009		
CSPH, yes vs. no			1.75 (1.10 to 2.81)	0.016
Extent of PVTT: main trunck vs. left/right branch			1.81 (0.99 to 3.53)	0.043
Tumor differentiation: III–IV vs. II			3.93 (1.56 to 10.18)	0.017

*AFP* α-fetoprotein, *TBIL* total bilirubin, *CSPH* clinical significant portal hypertension, *PVTT* portal vein tumor thrombus

### Patients who survived > 2 years

Descriptive comparison between patients who survived > 2 years (n = 98) post-hepatectomy and those who died within 2 years (n = 291) after surgery revealed that survivors had a favorable pre-operative condition characterized by lower AFP, better pre-operative liver function, smaller tumor diameter and more favorable tumor differentiation (Table 4).

**Table 4:** Descriptive analysis of survivors versus non-survivors at 2 years

Variable	Alive (n = 98)	Died (n = 291)	*P*
Age, years	48.9 ± 11.1	47.7 ± 10.4	0.368
Sex			1.000
Female	11 (11.2%)	34 (11.7%)	
Male	87 (88.8%)	257 (88.3%)	
Viral marker			
HBsAg positive	87 (89.7%)	266 (92.4%)	0.541
Anti-HCV positive	0 (0.0%)	2 (0.7%)	1.000
AFP, μg/L	763 (1.2–138,410)	1210 (1.8–777,600)	0.012
AFP, Log 10 μg/L	2.88 (0.08–5.14)	3.08 (0.26–5.89)	0.012
TBIL, mg/dL	0.77 (0.26–1.87)	0.85 (0.30–7.54)	0.024
ALB, g/L	41.6 (17.2–67.2)	40.8 (17.8–65.3)	0.421
PT, s	12.1 (9.9–14.9)	12.4 (9.6–18.0)	0.054
ALT, U/L	42.8 (11.2–187.8)	43.0 (8.7–169.9)	0.875
PLT, 10^9^/L	172 (25.0–405)	151 (25.0–629)	0.148
WBC, 10^9^/L	5.57 (1.80–10.4)	5.44 (1.44–19.4)	0.672
Hemoglobin, g/L	140 (104–174)	141 (75.0–195)	0.984
Child–Pugh class			0.460
A	97 (99.0%)	283 (97.3%)	
B	1 (1.0%)	8 (2.7%)	
Radiologic ascites			0.110
No	94 (95.9%)	262 (90.0%)	
Mild	4 (4.1%)	29 (10.0%)	
Gastroesophageal varices			0.155
No	88 (89.8%)	242 (83.2%)	
Yes	10 (10.2%)	49 (16.8%)	
Radiologic spleen length, cm	11.0 (6.3–16.4)	11.7 (6.3–20.2)	0.005
CSPH			0.010
No	60 (61.2%)	138 (47.4%)	
Mild	36 (36.7%)	124 (42.6%)	
Severe	2 (2.1%)	29 (10.0%)	
Type of hepatectomy			0.130
En bloc resection	56 (57.1%)	193 (66.3%)	
Non-en bloc resection	42 (42.9%)	98 (33.7%)	
Operating time, h	2.0 (0.45–20.8)	2.0 (0.45–10.3)	0.488
Hilar clamping time, min	21.0 (0–58.0)	20.0 (0–83.0)	0.334
Intraoperative blood loss, mL	325 (100–3200)	400 (0–45,000)	0.038
Transfusion			0.409
No	73 (74.5%)	202 (69.4%)	
Yes	25 (25.5%)	89 (30.6%)	
Surgical margins			0.690
R0	81 (82.7%)	227 (78.0%)	
R1	2 (2.0%)	9 (3.1%)	
Unconfirmed	15 (15.3%)	55 (18.9%)	
Extent of PVTT			0.115
Left branch	22 (22.4%)	69 (23.7%)	
Right branch	62 (63.3%)	154 (52.9%)	
Main trunck/contralateral branch	14 (14.3%)	68 (23.4%)	
Tumor number			0.781
Solitary	88 (89.8%)	266 (91.4%)	
Multiple	10 (10.2%)	25 (8.59%)	
Tumor diameter, cm	7.7 (2.0–18.2)	8.8 (0.4–24.0)	0.049
Cirrhosis			0.067
No	35 (35.7%)	74 (25.4%)	
Yes	63 (64.3%)	217 (74.6%)	
Tumor differentiation			0.007
II	11 (11.2%)	9 (3.09%)	
III	85 (86.7%)	277 (95.2%)	
IV	2 (2.1%)	5 (1.72%)	

*HBsAg* hepatitis B surface antigen, *anti-HCV* hepatitis C virus antibody, *AFP* α-fetoprotein, *TBIL* total bilirubin, *ALB* albumin, *PT* pro-thrombin time, *ALT* alanine aminotransferase, *PLT* platelet count, *WBC* white blood cell, *CSPH* clinical significant portal hypertension, *PVTT* portal vein tumor thrombus

Patients who survived for at least 2 years (n = 98) were compared with those who died within 2 years after surgery (n = 291), and results showed that the following factors were associated with death within 2 years after surgery (Additional file 1: Table S1). In multivariable analysis, AFP, per Log 10 μg/L increase (OR, 1.34; 95% CI 1.06 to 1.69; P = 0.034), clinically significant portal hypertension (CSPH) (OR, 1.75; 95% CI 1.10 to 2.81; P = 0.034), extent of PVTT (OR, 1.81; 95% CI 0.99 to 3.53; P = 0.043) and tumor differentiation III–IV (OR, 3.93; 95% CI 1.56 to 10.18; P = 0.017) were significantly associated with death within 2 years.

### Effects of AFP and TBIL on survival in cirrhosis context

**Fig. 2:**
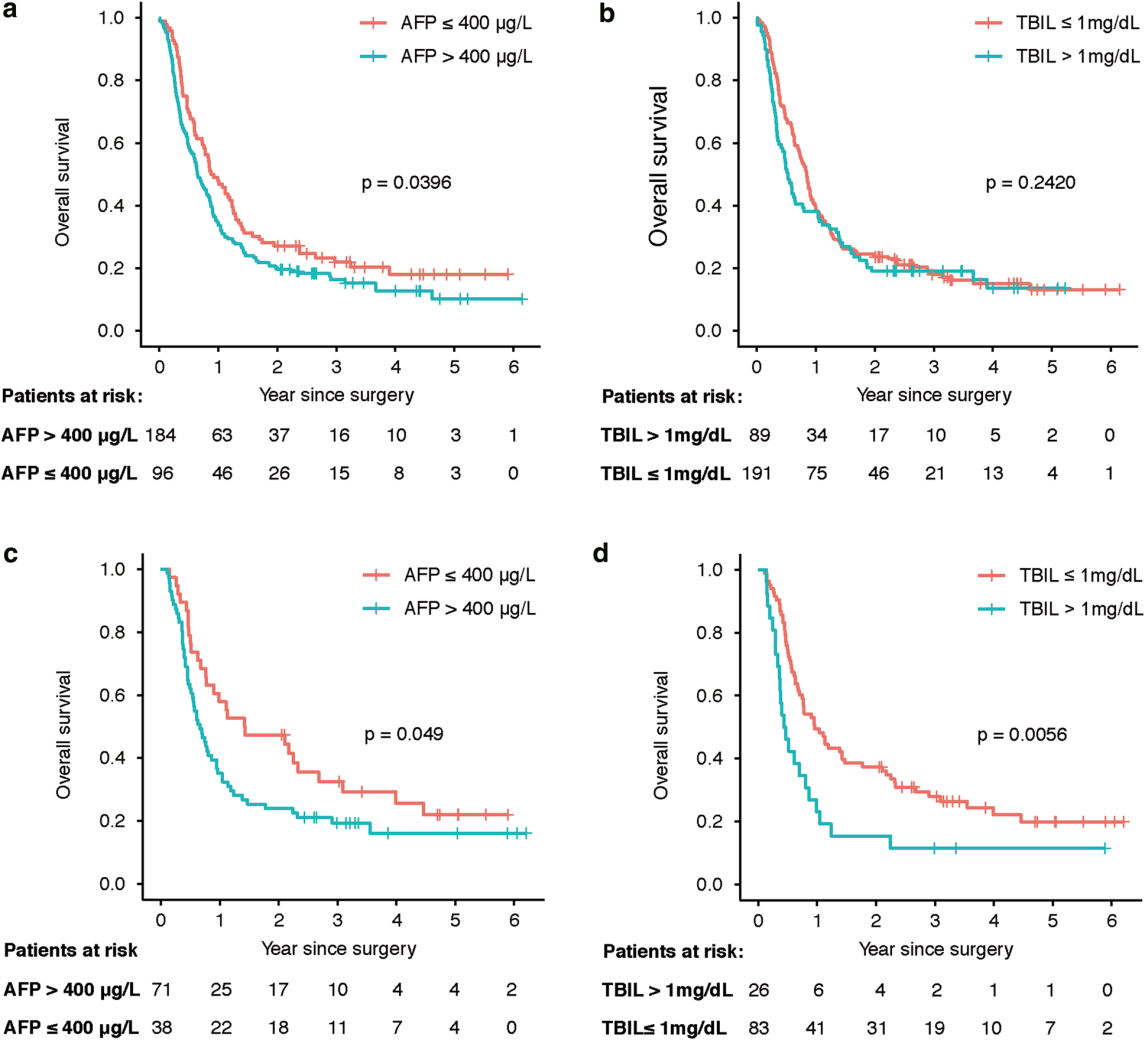
Survival curve of OS for cirrhosis subgroup. **a** Survival curve for AFP level in patients with cirrhosis. **b** Survival curve for TBIL level in patients with cirrhosis. **c** Survival curve for AFP level in patients without cirrhosis. **d** Survival curve for TBIL level in patients without cirrhosis. *OS* overall survival

Survival was compared across subgroup based on patients’ cirrhosis context. In the subset of patients with cirrhosis, the 1-, 3- and 5-year OS rate for patients with low or high serum AFP level was 47.9%, 22% and 18% or 34.2%, 16.3% and 10.2%, respectively (P = 0.0398, Fig. 2a), while for patients with low or high serum TBIL level it became 39.3%, 17.9% and 13.2% or 38.2%, 19.1% and 13.6%, respectively (P = 0.242, Fig. 2b). In the subset of patients without cirrhosis, the 1-, 3- and 5-year OS rate for patients with low or high serum AFP level was 57.9%, 32.6% and 22% or 35.20%, 19.4% and 16.1%, respectively (P = 0.049, Fig. 2c), while for patients with low or high serum TBIL level it became 49.4%, 28% and 19.9% or 23.1%, 11.5% and 11.5%, respectively (P = 0.006, Fig. 2d).

## Discussion

The presence of PVTT in HCC patients is regarded as indication of an advanced stage, and LR is not recommended [14, 15]. Clinical outcome of patients who require surgical treatment of PVTT predominantly depends on appropriate patient selection, which is complex and involved in several factors. Some patients may not receive liver resection, although they may benefit from it, whereas others receive liver resection but disease still progresses rapidly. Previous studies [11, 12] have reported that physicians often consider locations of PVTT and tumor for HCC patients with PVTT, and several staging methods [5–7, 15] have been developed to help predict survival and guide treatment in patients with HCC and PVTT. However, there is still a lack of data for guiding surgeons and cancer patients to select appropriate treatment for HCC patients with PVTT.

In this study, we investigated pre-operative factors associated with short-term or long-term survival in a prospectively observed cohort of 457 patients who underwent surgery for treatment of HCC and PVTT. Our results suggested that patients who died within 3 months after surgery were significantly associated with preoperatively worsened liver function, such as total bilirubin or ascites, compared with those who survived beyond the first 3 months, while patients surviving for > 2 years after surgery were associated with CSPH and factors of tumor aggressive nature, such as tumor differentiation, AFP and extent of PVTT.

Our results of analysis for short-term survival patients indicated that patients with poor pre-operative liver function were not suitable for liver resection, as our results clearly showed worse survival for patients with unfavorable pre-operative parameters, including AFP, TBIL and radiologic ascites. Recovery of liver dysfunction should be recommended first. Radiologic ascites become a predominant prognostic variable for decision making. In clinical situation, although for patients with Child A class, ascites is common. This implied we can more accurately select candidates for liver resection, even for patients with Child A class. There is a research reporting that CTP class can affect the survival of HCC patients with PVTT [16], which is similar to our findings.

Longer-term survivors often did not have hepatic decompensation, while the patients who had CSPH always exhibited worse long-term outcome. There is a similar research reporting that the risk of 3-year and 5-year mortality can be increased by the presence of CSPH, which also increases the risk of postoperative clinical decompensation [17]. The factors also indicated that tumor aggressive nature, such as tumor differentiation, AFP and extent of PVTT, were tightly associated with long-term survival of patients. Of the four factors, CSPH, AFP and extent of PVTT can be obtained preoperatively, and according to this we can make recommendation that resection treatment was not suitable for patients with high AFP, PVTT in the main portal vein, and CSPH. The factor tumor differentiation is a pathological indicator and can only be obtained after surgery, which may provide reference for whether adjuvant therapy after surgery should be given or not.

Our result showed that serum AFP level was a key factor of both short-term and long-term survivals for patients with PVTT. We further investigated the staining pattern of AFP in both HCC tissues and peritumoral tissues according to immunohistochemical analysis (Additional file 2: Supplementary method). The immunostaining results showed the expression of AFP in short-term survivors was higher than that in long-term survivors, which were consistent with the serum AFP results of this study (Additional file 3: Figure S1). Furthermore, the expression of AFP was elevated in tumor tissues, while it was decreased in peritumoral tissues, which was similar to previous study [18].

Liver cirrhosis is common in patients with advanced HCC [19], and a large proportion of cirrhosis was also observed in patients with PVTT of this study. To evaluate whether the cirrhosis context was associated with the effect of risk factors on survival in long-term survivors, we conducted survival analysis for AFP and TBIL level in subset of cirrhosis. Survival analysis showed that AFP is a risk factor for prognosis in both patients with and without cirrhosis. However, TBIL was a risk factor only in patients without cirrhosis, and the effect of TBIL on prognosis was diminished in the context of cirrhosis. This results suggested that although cirrhosis was not a risk factor for prognosis in patients with PVTT, the existence of cirrhosis made some liver function factors, such as TBIL, no longer a risk factor for prognosis of long-term survivors. However, the presence of cirrhosis did not affect the characteristics of the tumor itself, such as AFP.

Although our current study showed consistency over multiple pre-operative predictors of improved survival for patients, several biases might be present. First, although each participating center provided up-to-date information on vital statistics, inaccuracies in the actual status of patients could not be ruled out as many patients were referred back to their own referring hospitals. As the chance of a dead patient had higher possibility of being lost during follow-up in the database than the opposite, this potential inaccuracy could lead to an overestimation of effect of the end points. Second, the majority of patients had a background of HBV infection, which might be not suitable for patients with other background. The common factor for all patients was the need of surgical treatment. For each patient, indication to proceed with surgery was based on the need to alleviate the symptoms of portal hypertension and a presumed life expectancy of > 3 months. All these factors could be biased by the experience and preference of the anesthesiologist, surgeon, oncologist, institutional preference and patient wishes.

In conclusion, this large prospective cohort study strongly suggested that survival depended on general pre-operative status for liver resection of HCC and PVTT. In particular, serum AFP level, TBIL level, and radiologic ascites were significantly and independently associated with poor short-term survival (< 3 months), while serum AFP level, tumor differentiation, CSPH, and extent of PVTT were significantly and independently associated with longer-term survival.

## Methods

### Patients

A total of 457 consecutive HCC patients with PVTT who underwent LR at the Eastern Hepatobiliary Surgery Hospital (EHBH) from January 2008 to December 2010 were enrolled in the present study, and their electronic medical records were systematically reviewed. All patients were histologically diagnosed as HCC. Our retrospective analysis of data was approved by the Institutional Ethics Committee of the EHBH. Informed consent was obtained from all the patients before surgery.

All surgical team members adhered to the principle of performing LR for individuals whose life expectancy should be no less than 3 months.

### LR procedure

Based on the location and extent of PVTT, thrombectomy was carried out. The en bloc resection was considered if there was a sufficient safety margin between its root and the tip of the thrombus. Once the PVTT protruded into the main portal vein beyond the resection line, PVTT was removed from the opened stump of the portal vein. The main portal trunk was exposed and clamped from distal to the PVTT if the PVTT extended into the main portal trunk and its primary branches on both sides. An incision was made at the bifurcation of the right and left portal veins to extract the PVTT. The incision was flushed with normal saline, and the presence of PVTT was confirmed. Subsequently, the stump was closed by a continuous suture.

The commonly used methods of surgical resection are as follows. (1) Segmental hepatectomy: HCC is confined to the segment of liver, and PVTT in the segmental branches of portal vein can be excised in the same segment, which is suitable for type I and partial II PVTT patients. (2) Hemi-hepatectomy: HCC is located on the left or right lobe of liver, and PVTT in the first branch of portal vein can be excised in the same half-liver, which is suitable for partial patients with type II and III PVTT. (3) Hepatectomy plus thrombectomy: PVTT extends to the bifurcation of the right and left portal veins, or to the main portal vein, which is suitable for patients with type III PVTT. In the hepatectomy for HCC, concomitant thrombectomy is performed with temporary occlusion of the portal veins before the tumor thrombus is removed from the vessels. An incision of portal vein is carried out at the bifurcation of the right and left portal veins, and then PVTT is extracted from the incision in the portal vein. If there is a sufficient safety margin between its root and the tip of the thrombus for portal vein ligation, the en bloc resection, including the bifurcation with or without the main and/or contralateral portal veins, can be used for HCC patients with PVTT. (4) Portal vein resection and reconstruction: PVTT invades the main portal vein wall and is difficult to be removed, which is suitable for some patients with type III and IV PVTT. In the hepatectomy, the invaded main portal vein may be removed together with PVTT, and the 6/0 Prolene continuous suture with a 1-cm growth factor was used in direct end-to-end portal vein anastomosis. If the resected portal vein is too long, the autologous vein or artificial blood vessel can be used as graft. Hepatic artery and portal vein chemotherapy pump implantation are suitable for the patients with PVTT removed in the hepatectomy, and the pathway for the postoperative regional chemotherapy is reserved by hepatic artery and portal vein chemotherapy pump during the operation. Direct suction or back-bleeding is conducted if there is any residual tumor thrombus in the branch of portal vein. Residual tissue containing potential tumor thrombus should be removed by flushing the portal vein lumen with water and heparinized saline.

### Definitions of resection type

R0 resection was defined by the absence of microscopic tumor invasion of the resection margin; R1 resection was defined as a complete macroscopic resection with a positive pathological margin; and R2 resection was defined as a macroscopically positive margin.

### Classification of PVTT

The criteria used by Chen et al. [9] were employed in the present study, and the types of PVTT were classified into three subgroups as follows: (a) type A was defined as PVTT in the main portal vein; (b) type B was defined as PVTT in the first-order portal vein branch (the right or left portal vein); and (c) type C was defined as PVTT in the second- or lower-order portal vein branch (segmental branches of portal vein or higher).

### Follow-up and endpoints

Survival at 3 months post-operation was the primary outcome, and survival at 2 years post-operation was the secondary outcome. To ensure reliability of primary and secondary outcomes, all the case records were reviewed by participating investigators after closure of the database with special emphasis on vital statistics, no matter dead or alive for each patient. OS was also calculated from the date of surgery to the date of patient death or last follow-up.

### Statistical analysis

Statistical analysis was performed using the R statistical package v.3.2.1 (http://www.r-project.org/). Continuous data were expressed as mean ± SD or median (range), and comparisons were conducted using the Student’s t-test or Mann–Whitney U test. The Pearson’s χ^2^ test or Fisher’s exact test was used to compare categorical data. Data distributions before analysis of results and calculations of statistical parameters were reviewed. Factors associated with death within 3 months were assessed by univariable and multivariable logistic regression analyses of cases without missing data since hepatectomy and factors associated with survival after surgery for > 2 years. Survival curves were established using the Kaplan–Meier method and compared using the log-rank test. P values < 0.05 were considered statistically significant.

## Additional files

**Additional file 1: Table S1.** Univariable logistic regression analysis exploring factors associated with death within 3 or 24 months after hepatectomy.

**Additional file 2.** Supplementary method.

**Additional file 3: Figure S1.** AFP expression in HCC tissue, paired peritumoral tissue, and normal liver tissue. (A) AFP expression in control liver tissue, adjacent tissue < 1cm from tumor, tissue at operative site (> 1 cm from tumor or more), and tumor tissue for long-term and short-term survivors. Tissue specimens were immunostained with antibody directed against AFP (GB11287, Servicebio, Shanghai, China). Representative examples are shown. Panel B-C show AFP expression in control livers of non-HCC patients. Panels D-F show AFP expression in long term HCC patient of adjacent tissue < 1cm from tumor (D), tissue at operative site (E), and tumor tissue (F). Panels G-I show AFP expression in short term HCC patient of adjacent tissue < 1cm from tumor (G), tissue at operative site (H), and tumor tissue (I).

## Abbreviations

PVTT: portal vein tumor thrombosis
HCC: hepatocellular carcinoma
LR: liver resection
OS: overall survival
AFP: alpha-fetoprotein
TBIL: total bilirubin
OR: odds ratio
CSPH: clinically significant portal hypertension
CTP: Child–Turcotte–Pugh
ECOG: electrocorticography
SD: standard deviation
