# Supplementary material for: Short-term and long-term outcomes of liver resection for HCC patients with portal vein tumor thrombus
Source: Cell Biosci. 2019 Mar 6;9:23. doi: 10.1186/s13578-019-0285-z (PMC6404349; doi:10.1186/s13578-019-0285-z)
Supplement: Supplementary file 1 — Additional file 1: Table S1. Univariable logistic regression analysis exploring factors associated with death within 3 or 24 months after hepatectomy. [file 13578_2019_285_MOESM1_ESM.docx]

| **Table S1. Univariable logistic regression analysis exploring factors associated with death within 3 or 24 months after hepatectomy** | | | | | |
| --- | --- | --- | --- | --- | --- |
| Variable | **Three month analysis** | |  | **Two year analysis** | |
|  | Odds Ratio (95% CI) | *P* |  | Odds Ratio (95% CI) | *P* |
| **Age**, years | 1.01 (0.98 to 1.04) | 0.438 |  | 0.99 (0.97 to 1.01) | 0.350 |
| **Sex**, male | 0.90 (0.38 to 2.48) | 0.817 |  | 0.96 (0.45 to 1.94) | 0.921 |
| **HBsAg**, positive vs. negative | 3.72 (0.78 to 88.71) | 0.116 |  | 1.40 (0.61 to 3.02) | 0.415 |
| **AFP**, Log 10 μg/L | 1.60 (1.15 to 2.25) | 0.006 |  | 1.34 (1.06 to 1.69) | 0.014 |
| **TBIL**, mg/dL | 1.94 (1.21 to 3.12) | 0.006 |  | 2.25 (1.09 to 4.65) | 0.028 |
| **ALB**, g/L | 1.01 (0.97 to 1.06) | 0.559 |  | 0.99 (0.95 to 1.02) | 0.538 |
| **PT**, seconds | 1.28 (0.99 to 1.65) | 0.059 |  | 1.22 (0.97 to 1.52) | 0.092 |
| **ALT**, U/L | 1.00 (0.99 to 1.01) | 0.617 |  | 1.00 (1.00 to 1.01) | 0.598 |
| **PLT**, 10^9^/L | 1.00 (1.00 to 1.00) | 0.637 |  | 1.00 (1.00 to 1.00) | 0.990 |
| **WBC**, 10^9^/L | 0.97 (0.84 to 1.12) | 0.665 |  | 1.06 (0.95 to 1.19) | 0.303 |
| **Hemoglobin**, g/L | 1.00 (0.99 to 1.00) | 0.703 |  | 1.00 (1.00 to 1.00) | 0.761 |
| **Child-Pugh class**, B vs. A | 2.43 (0.32 to 10.74) | 0.334 |  | 2.44 (0.43 to 61.96) | 0.364 |
| **Radiologic ascites**, positive vs. negative | 3.72 (1.59 to 8.15) | 0.003 |  | 2.51 (0.95 to 8.86) | 0.064 |
| **Gastroesophageal varices**, yes vs. no | 1.48 (0.69 to 2.95) | 0.305 |  | 1.76 (0.88 to 3.84) | 0.111 |
| **Radiologic spleen length**, cm | 1.15 (1.02 to 1.30) | 0.024 |  | 1.15 (1.04 to 1.28) | 0.009 |
| **CSPH**, yes vs. no | 1.70 (0.94 to 3.13) | 0.080 |  | 1.75 (1.10 to 2.81) | 0.019 |
| **Type of hepatectomy**, non-en bloc vs. en bloc | 0.70 (0.36 to 1.30) | 0.261 |  | 0.68 (0.42 to 1.09) | 0.106 |
| **Operating time**, hour | 0.98 (0.81 to 1.18) | 0.829 |  | 0.94 (0.80 to 1.11) | 0.459 |
| **Hilar clamping time**, min | 1.01 (0.98 to 1.03) | 0.507 |  | 0.99 (0.97 to 1.01) | 0.468 |
| **Intraoperative blood loss**, mL | 1.00 (1.00 to 1.00) | 0.582 |  | 1.00 (1.00 to 1.00) | 0.298 |
| **Transfusion**, yes vs. no | 1.28 (0.67 to 2.36) | 0.442 |  | 1.28 (0.77 to 2.19) | 0.344 |
| **Surgical margins** |  |  |  |  |  |
| R0 | Ref. | Ref. |  | Ref. | Ref. |
| R1 | 0.58 (0.02 to 2.97) | 0.579 |  | 1.52 (0.37 to 11.09) | 0.593 |
| Unconfirmed | 0.90 (0.37 to 1.91) | 0.794 |  | 1.30 (0.71 to 2.51) | 0.406 |
| **Extent of PVTT**,  main trunck vs. left/right branch | 1.28 (0.63 to 2.46) | 0.486 |  | 1.81 (0.99 to 3.53) | 0.054 |
| **Tumor number**, multiple vs. solitary | 1.05 (0.34 to 2.58) | 0.930 |  | 0.82 (0.39 to 1.87) | 0.622 |
| **Tumor diameter**, cm | 1.08 (1.02 to 1.16) | 0.015 |  | 1.06 (1.00 to 1.13) | 0.045 |
| **Cirrhosis**, yes vs. no | 1.73 (0.87 to 3.77) | 0.125 |  | 1.63 (0.99 to 2.66) | 0.054 |
| **Tumor differentiation**, III-IV vs. II | 2.65 (0.54 to 63.75) | 0.279 |  | 3.93 (1.56 to 10.18) | 0.004 |

**Abbreviations:** HBsAg, hepatitis B surface antigen; Anti-HCV, hepatitis C virus antibody; AFP, α-fetoprotein; TBIL, total bilirubin; ALB, albumin; PT, pro-thrombin time; ALT, alanine aminotransferase; PLT, platelet count; WBC, white blood cell; CSPH, clinical significant portal hypertension; PVTT, portal vein tumor thrombus.
