# Supplementary material for: Short-term and long-term outcomes of liver resection for HCC patients with portal vein tumor thrombus
Source: Cell Biosci. 2019 Mar 6;9:23. doi: 10.1186/s13578-019-0285-z (PMC6404349; doi:10.1186/s13578-019-0285-z)
Supplement: Supplementary file 2 — Additional file 2. Supplementary method. [file 13578_2019_285_MOESM2_ESM.docx]

**Additional file 2**

**Method**

***Samples and immunohistochemistry***

Paraffin-embedded surgical specimens were obtained from 60 HCC patients studied in this study, of which 30 were patients with short-term survival (<2 year) and 30 with long-term survival (> 2year). For each patient, specimens of tumor tissue, adjacent tissue (< 1cm from tumor), and tissue at the surgical margin (> 1cm or more from tumor) were collected. Control liver tissues were obtained from 10 patients with hepatic hemangioma.

These immunostaining experiments were performed as previously described [1]. The anti-AFP antibodies (GB11287, Servicebio, Shanghai, China) were used immunostaining. The intensity of positive staining of AFP was measured through Image-Proplus 6.0 software (Media Cybernetics, Inc., Rockville, MD, USA). For each sample, four representative images of the liver tissue were analysed using Image-pro plus 6.0 .Each photo was analyzed using Image-Pro Plus 6.0 software to obtain the Integrated optical density (IOD) and the area of the tissue (Area), and the value of IOD/Area was calculated. Statistical analysis was carried out using R software v.3.2.1 (http://www.r-project.org/). The Mann-Whitney test was used to analyze and the results expressed as quantiles interval. Differences were considered significant when the P value was < 0.05.

**Results:**

**Table S1.** Univariable logistic regression analysis exploring factors associated with death within 3 or 24 months after hepatectomy.

**Figure S1.** AFP expression in HCC tissue, paired peritumoral tissue, and normal liver tissue.

**Reference:**

[1] Hou, Y., Zou, Q., Ge, R., Shen, F. & Wang, Y. The critical role of CD133(+)CD44(+/high) tumor cells in hematogenous metastasis of liver cancers. Cell Res. 22, 259-272 (2012).

**Figure legend:**

**Figure S1.** AFP expression in HCC tissue, paired peritumoral tissue, and normal liver tissue. (A) AFP expression in control liver tissue, adjacent tissue < 1cm from tumor, tissue at operative site (> 1 cm from tumor or more), and tumor tissue for long-term and short-term survivors. Tissue specimens were immunostained with antibody directed against AFP (GB11287, Servicebio, Shanghai, China). Representative examples are shown. Panel B-C show AFP expression in control livers of non-HCC patients. Panels D-F show AFP expression in long term HCC patient of adjacent tissue < 1cm from tumor (D), tissue at operative site (E), and tumor tissue (F). Panels G-I show AFP expression in short term HCC patient of adjacent tissue < 1cm from tumor (G), tissue at operative site (H), and tumor tissue (I).
