# Supplementary figures and images for: Short-term and long-term outcomes of liver resection for HCC patients with portal vein tumor thrombus
Source: Cell Biosci. 2019 Mar 6;9:23. doi: 10.1186/s13578-019-0285-z (PMC6404349; doi:10.1186/s13578-019-0285-z)

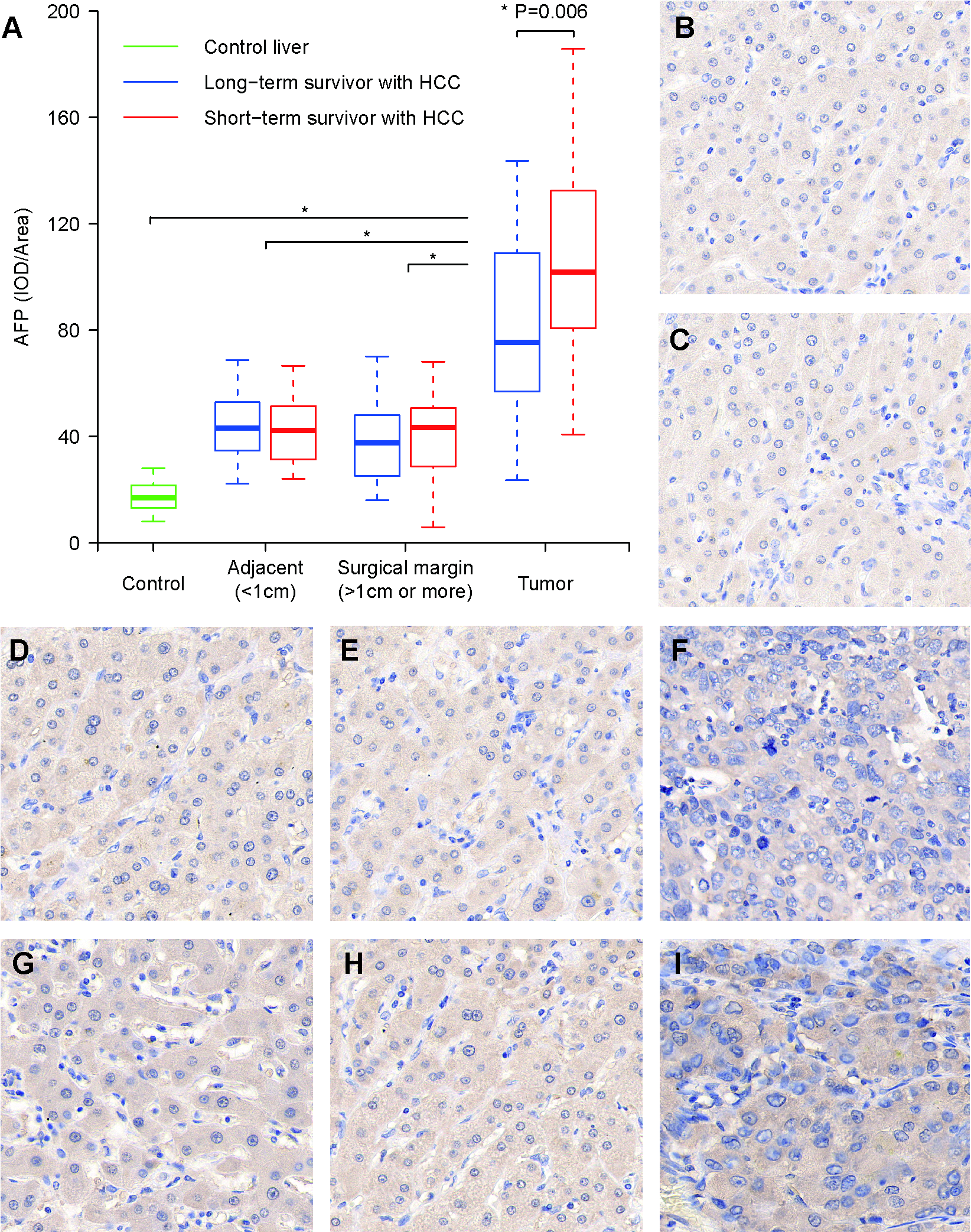

Supplement: Supplementary file 3 — Additional file 3: Figure S1. AFP expression in HCC tissue, paired peritumoral tissue, and normal liver tissue. (A) AFP expression in control liver tissue, adjacent tissue < 1cm from tumor, tissue at operative site (> 1 cm from tumor or more), and tumor tissue for long-term and short-term survivors. Tissue specimens were immunostained with antibody directed against AFP (GB11287, Servicebio, Shanghai, China). Representative examples are shown. Panel B-C show AFP expression in control livers of non-HCC patients. Panels D-F show AFP expression in long term HCC patient of adjacent tissue < 1cm from tumor (D), tissue at operative site (E), and tumor tissue (F). Panels G-I show AFP expression in short term HCC patient of adjacent tissue < 1cm from tumor (G), tissue at operative site (H), and tumor tissue (I). [file 13578_2019_285_MOESM3_ESM.tif]
